# Evaluating the impacts of critical time intervention implementation on health service utilization among homeless-experienced veterans: a retrospective observational study

**DOI:** 10.1186/s12913-026-14942-z

**Published:** 2026-07-03

**Authors:** Kimberly S. Clair, Kristina M. Cordasco, Alec Chapman, David A. Ganz, Nicholas Jackson, Ann E. Montgomery, Richard Nelson, Melissa Y. Wei, Erin P. Finley, Sonya Gabrielian

**Affiliations:** 1 Center for the Study of Healthcare Innovation, Implementation & Policy (CSHIIP), VA Greater Los Angeles Healthcare System, 11301 Wilshire Blvd, Los Angeles, CA 90073 USA; 2 David Geffen School of Medicine, University of California Los Angeles, 757 Westwood Plaza, Los Angeles, CA 90095 USA; 3 George E. Wahlen Department of Veterans Affairs Medical Center, 500 Foothill Boulevard, Salt Lake City, UT 84148 USA; 4 University of Utah, 201 Presidents Circle, Salt Lake City, UT 84112 USA; 5 Veterans Health Administration, 700 19th Street South, Birmingham, AL 35233 USA; 6 Departments of General & Hospital Medicine & Psychiatry, University of Texas Health Science Center, 7703 Floyd Curl Drive, San Antonio, TX 78229 USA

**Keywords:** Homelessness, Veteran health, Health care access

## Abstract

**Background:**

Critical Time Intervention (CTI) is an evidence-based, time-limited case management practice that improves housing outcomes among homeless-experienced adults. While CTI’s effect on housing outcomes has been well-studied, its impacts on health services utilization are less clear. In the context of a U.S Department of Veterans Affairs (VA) CTI implementation effort, we assessed relationships between CTI implementation, including fidelity, and Veterans’ VA health services utilization.

**Methods:**

We conducted a retrospective observational study of 9,051 Veterans who received services, 10/1/2019 to 5/1/2025, from one of 156 case management sites that worked with VA; 32 of these sites implemented CTI. Within the 18 implementing CTI sites that completed fidelity ratings, we compared health services utilization for Veterans (n = 2,022) at sites with adequate (n = 12 sites) vs. inadequate (n = 6 sites) fidelity. We performed multivariate regression using generalized estimating equations with clustering by site, adjusting for Veterans’ demographics and diagnoses, and VA facility complexity. Regression models identified the impacts of CTI (versus non-CTI), and fidelity (adequate versus inadequate), on health service utilization; we used negative binomial regression for count outcomes (number of primary care, emergency department [ED], behavioral health, homeless services, outpatient medical-surgical visits) and logistic regression for binary outcomes (presence of inpatient mental health, or medical-surgical hospitalizations).

**Results:**

CTI Veterans had lower rates of utilization across all service types compared to non-CTI, including primary care (IRR = 0.98, 95% CI: 0.97–0.98), ED (IRR = 0.93, 95% CI: 0.90–0.95), homeless services (IRR = 0.91, 95%CI: 0.89–0.93) and hospitalizations (AOR = 0.79, 95%CI: 0.68–0.91). Among CTI Veterans, Veterans at sites with adequate fidelity had more primary care use (IRR = 1.04, 95% CI: 1.02–1.07), less ED use (IRR = 0.84, 95% CI: 0.80–0.90) and more homeless service use (IRR = 1.10, 95% CI: 1.05–1.15).

**Conclusions:**

CTI Veterans had lower service utilization rates compared to non-CTI Veterans. However, adequate CTI fidelity is associated with increased primary care use and decreased ED use; this finding may be due to high-quality CTI implementation and/or site-level characteristics that enabled better CTI fidelity. Ensuring fidelity to CTI implementation may hold value in promoting optimal care linkages for homeless-experienced Veterans and other clients with high needs.

## Background

The U.S. Department of Housing and Urban Development (HUD) estimated just under 33,000 Veterans experiencing homelessness in 2024, comprising 5% of all adults experiencing homelessness [1]. Veteran homelessness has been declining since 2009 [1], due in large part to VA’s multi-pronged, robust approach to addressing Veteran homelessness, with services ranging from street outreach, transitional housing, residential rehabilitation programs, homeless-tailored healthcare teams, and a large permanent supportive housing program [2]. Yet, one in ten Veterans is still estimated to experience homelessness in their lifetime [3], with significant, negative effects on health. Homeless-experienced adults have a higher prevalence of medical illness and higher age-adjusted mortality compared to housed adults [4–6] while homeless Veterans have higher rates of severe mental illness, post-traumatic stress disorder (PTSD), depression, and suicidality compared to non-Veteran adults experiencing homelessness [3]. Additionally, homeless-experienced adults face numerous barriers to receiving care, including disengagement from primary care [7] and disruptions in care continuity [8–10], which contribute to high rates of acute care use [11, 12].

Critical Time Intervention (CTI) is an evidence-based, time-limited (6–9 month) case management practice designed to support care continuity for homeless-experienced adults during transitions from institutional settings to the community [13, 14]. By providing additional support during these critical transitional periods, CTI may improve regular healthcare engagement and reduce utilization of acute or crisis-related services. However, though there is robust evidence that CTI improves housing outcomes among adults with homeless experience [13, 15], the relationships between CTI and health services utilization are not well understood.

While CTI was associated with decreased psychiatric admission rates in several studies [16, 17], its impacts on utilization of other health services, including primary care, emergency department (ED), mental health, and substance use disorder services, are inconclusive. For example, participants in a “brief” (3 month) CTI intervention following inpatient hospitalizations showed greater continuity of mental health and substance use care compared to participants who did not receive CTI [18]; however, no significant differences between CTI and non-CTI groups were observed in mental health and substance use services utilization among adults with psychiatric readmissions [19]. In a systematic review, CTI was found to have a positive impact on housing and health services utilization within the United States but had inconsistent effects on these outcomes when implemented outside of the United States [20].

Moreover, relationships between CTI and health services utilization may be affected by the extent to which CTI is implemented as intended (CTI fidelity) [21]. Incorporating information on CTI fidelity when evaluating the impacts of CTI on health services utilization may provide a more nuanced understanding of these relationships. Yet few studies in the literature have measured CTI fidelity or explored its associations with clients’ service use.

The VA currently awards $30 million every two years to fund the Grant and Per Diem (GPD) case management “Aftercare” program, with the aim of supporting Veterans’ transitions to independent living and preventing returns to homelessness [22]. In this program, VA contracts with non-VA community-based organizations to provide homeless-experienced Veterans with 6 months of case management during transitions to permanent housing. However, no specific case management approach is required, which may produce considerable variation in case management implementation and quality across more than one hundred funded programs. Implementing evidence-based, standardized case management in this context has the potential to improve the quality and efficacy of this high-value GPD program that has been mandated by congressional legislature [23].

To this end, we implemented CTI in 32 Aftercare sites [23]; though the VA had invested in CTI training across a number of its homeless services, this was to our knowledge the first concerted, longitudinal initiative to implement CTI in a homeless program. As part of this effort, among Veterans enrolled in VA healthcare, we evaluated VA health services utilization among Veterans who received Aftercare at sites that implemented CTI (“CTI Veterans”), compared to Veterans who received Aftercare at usual practice control sites that did not implement CTI (“non-CTI Veterans”). Among CTI Veterans, we examined how fidelity to CTI at their site was associated with health services use. Though the literature is mixed about the impact of CTI on health services utilization, we anticipated that, compared to non-CTI Veterans, CTI Veterans would have more use of ambulatory care (primary care, behavioral health, homeless services, medical-surgical visits) and decreased use of acute care services (ED, inpatient). Among CTI Veterans, we hypothesized that those at sites with adequate implementation fidelity would have higher rates of ambulatory care use and decreased use of acute care compared to Veterans at sites with inadequate CTI fidelity.

## Methods

### Study design and framework

We conducted a retrospective longitudinal study with a 12-month follow-up (one point of observation per Veteran at the end of 12 months, looking back on the prior 12 months). Our study was informed by the Behavioral Model for Vulnerable Populations [24], which identifies health behaviors (service utilization) as a function of predisposing characteristics (e.g., demographics), enabling factors (e.g., primary care access, CTI, fidelity to CTI), and evaluated need (e.g., diagnoses). We adapted this model to identify multi-level factors impacting health services utilization, including the aforementioned individual- and facility-level factors (e.g., VA facility complexity). All study procedures were reviewed by VA’s Central Institutional Review Board (IRB) and designated as non-research.

### Study sample

**Fig. 1:**
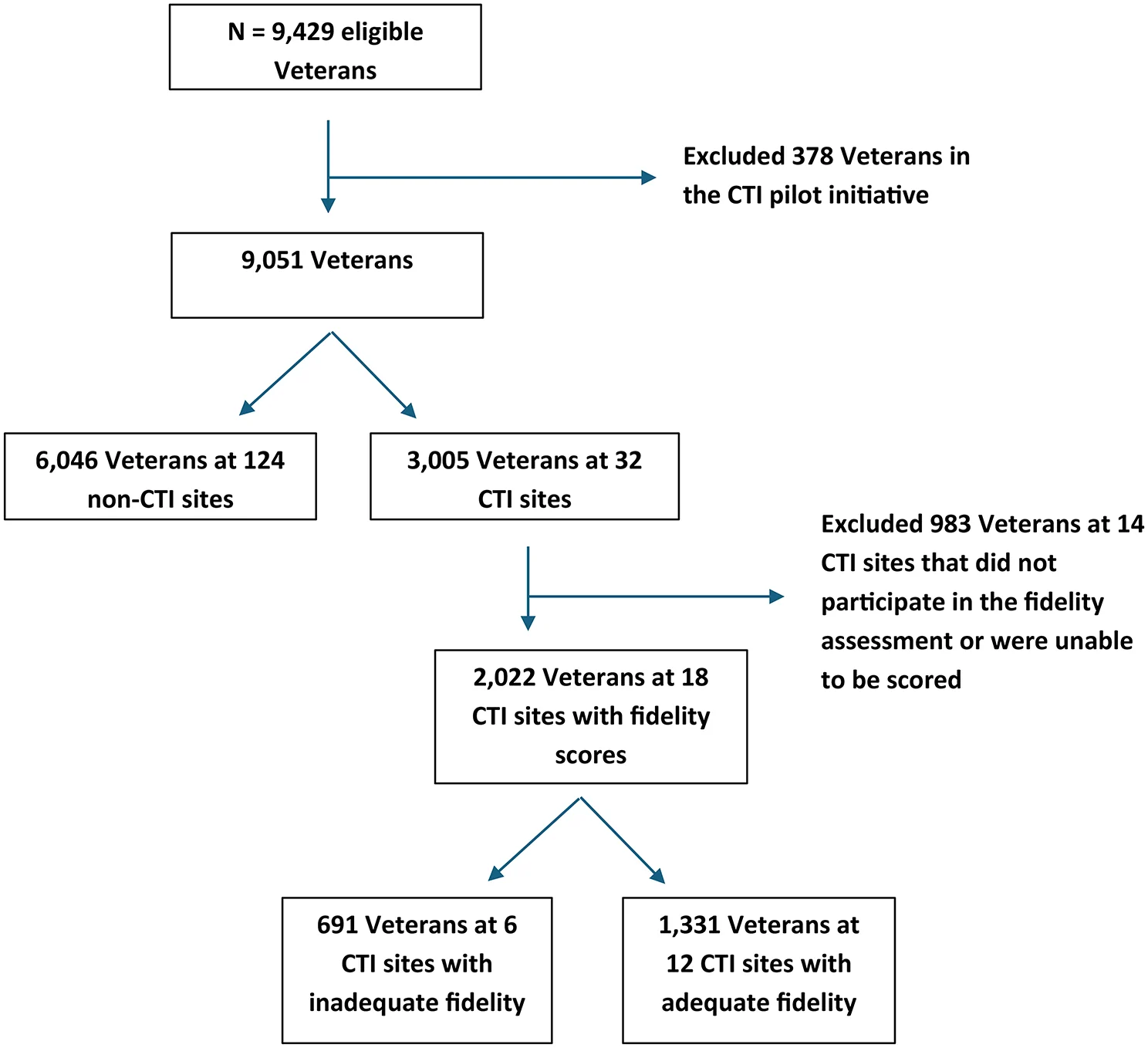
Participant Inclusion Criteria

Our sample included Veterans who participated in the Aftercare program, across its 156 sites, between 10/1/2019 and 5/1/2025 (*n* = 9,429). Our analytic sample (*n* = 9,051) excluded Veterans who participated in a pilot initiative to implement CTI in Los Angeles (*n* = 378, Fig. 1) [25]. We included 3,005 Veterans who received CTI and 6,046 Veterans who did not. Sites were selected to receive CTI implementation by VA regional homeless program leads; 32 sites implemented CTI and 124 did not. The basis for selection was twofold; while some sites were perceived as having high levels of readiness for CTI implementation, others were perceived as having suboptimal Veteran outcomes and in need of concerted CTI implementation efforts.

CTI was implemented in three successive cohorts of 10–11 sites/each, across 32 total implementing sites. Veterans were enrolled in CTI once implementation began at their site; thus, some “non-CTI Veterans” received services at sites that started CTI implementation after the Veterans entered the program. For all analyses, Veterans were classified as either “CTI” if they were exposed to CTI at any point during the three implementation cohorts, or “non-CTI” if they were not.

For analyses comparing Veterans at our 32 implementation sites by CTI fidelity outcomes, we additionally excluded Veterans at sites that did not participate in the fidelity assessment process or in which their fidelity was unable to be scored (e.g., due to inadequate documentation, *n* = 983 Veterans at 14 sites). Fidelity scoring procedures are described below. Fidelity ratings were dichotomized as adequate versus inadequate; we compared a sample of 1,331 Veterans at sites with adequate fidelity and 691 Veterans at sites with inadequate fidelity.

### Data sources

Data were obtained from VA’s Homeless Program Office and VA’s Corporate Data Warehouse (CDW). We obtained Veterans’ identifiers, including social security numbers (SSN) and Aftercare program information, from a roster maintained by VA’s GPD Aftercare program; the roster included Aftercare site and program entry and exit dates. Using SSN, we linked Aftercare program data to CDW to obtain demographics, diagnoses associated with health services encounters, and health services utilization data. CTI fidelity assessment procedures are detailed elsewhere [26]; in short, at 12 months after the implementation’s launch, we integrated data from a CTI implementation self-assessment designed to enhance provider practice and 90-minute videoconferences with case managers to collaboratively review charts from at least two “exemplar cases” (randomly selected by our fidelity assessors). Our assessor team included a clinical psychologist, a physician, and a masters’ level research assistant trained in CTI and supervised by the two clinician assessors. We used these data to note whether there was adherence to each of 7 fidelity domains [26]: decreasing intensity of case management; termination and transfer-of-care practices; duration of case management; use of case management phases; use of focus areas in each phase; caseload size; and clinical supervision practices. We dichotomized sites as having “adequate” or “inadequate” CTI fidelity, with adherence to a majority (≥ 4) of domains considered adequate. Additionally, we obtained data on VA site “complexity,” a designation of resource availability at each VA healthcare facility, which is categorized as levels of care offered [27], from the VA Support Service Center [28].

### Measures

#### Individual-level factors

##### Predisposing characteristics

Veteran sex (assigned at birth) was identified from CDW: male or female. Veteran age was identified from CDW at time of Aftercare program entry date and constructed as a continuous variable. Race/ethnicity was defined as a 5-category variable, including Non-Hispanic White, Non-Hispanic Black, Hispanic, Non-Hispanic Other Race (including Asian, American Indian and Alaska Native [AIAN], Native Hawaiian or Pacific Islander [NHOPI], and multi-racial individuals), and Non-Hispanic Unknown Race. Veterans who identified as Hispanic in addition to any racial group were coded as Hispanic. Marital status was defined as: separated/divorced, married, never married/single, widowed, unknown. Veterans’ geographic residence was categorized as a binary variable (urban vs. rural/highly rural/islands), which was obtained from geocoded patient location data found in CDW, and classified by a VA rurality indicator derived from the Rural-Urban Commuting Area (RUCA) codes [29]. Demographic data were identified at time of Aftercare entry date.

##### Enabling factors

To assess factors that enable healthcare utilization, we evaluated whether participants were empaneled in a patient-aligned care team (PACT, VA’s patient-centered medical home model) or a homeless patient-aligned care team (HPACT, tailored for homeless-experienced Veterans) in the year prior to Aftercare program entry, as Veterans with prior PACT or HPACT empanelment would typically experience fewer barriers to receiving continuing ambulatory care. We also considered *receipt of CTI* (received Aftercare at a site with CTI implementation vs. one without CTI implementation) and *fidelity to CTI* (adequate vs. inadequate) as binary enabling factors. Given CTI’s focus on establishing linkages to health services that can engage Veterans beyond the duration of time-limited case management, we hypothesized that CTI would assist Veterans in overcoming barriers to receiving ambulatory care.

##### Evaluated need

Need was defined via the proxy of Veterans’ medical, mental health, and substance use disorder (SUD) diagnoses. To assess multi-morbidity, we applied the Multimorbidity-Weighted Index (MWI), a validated measure that weights chronic conditions by their association with physical functioning, as described previously [30, 31] and implemented across VAs in the Care Assessment Need (CAN) 3.0 score [32, 33]. Chronic conditions were identified using International Classification of Diseases (ICD), Ninth and Tenth Revision (ICD-9, ICD-10) diagnostic codes from inpatient admissions or outpatient encounters in the CDW during the ten years preceding Aftercare entry (index) date. In regression analyses, we examined MWI both with and without mental health and SUD diagnoses, with the latter specification used to isolate medical multimorbidity (“adapted MWI”). To identify the presence versus absence of recent mental health and SUD symptoms, we identified diagnoses in the year prior to Aftercare program entry using ICD-10 codes associated with inpatient admissions or outpatient visits. Mental health diagnoses included schizophrenia and other psychotic spectrum disorders, bipolar disorder, other psychotic disorders, major depressive disorder (MDD), anxiety and post-traumatic stress disorder (PTSD); we created a binary indicator of having at least one of these mental health diagnoses. Additionally, we identified the presence versus absence of SUD diagnoses in the year prior to Aftercare program entry, related to alcohol, opioid, cannabis, sedative, cocaine, other stimulant, inhalant, hallucinogen, and other psychoactive drugs. We created a binary variable to identify Veterans with any one of these SUD diagnoses. To identify the presence of a diagnosis, we counted at least two diagnosis records from outpatient visits or one record from inpatient admissions.

##### Health behaviors

Our primary outcomes of interest were counts of outpatient VA ambulatory care visits one year following Aftercare-program entry, including primary care, ED, behavioral health, homeless services, and specialty medical-surgical care. Behavioral health was defined as the combined count of outpatient mental health and outpatient SUD visits. In addition, we assessed whether Veterans had any hospitalizations, which we further subdivided into any psychiatric hospitalizations versus medical-surgical hospitalizations in the year post Aftercare-program entry using binary variables (yes/no).

#### Facility-level factors

##### Site complexity

We included VA facility complexity [28] at the VA facility where Veterans received their care to account for facility-level factors impacting utilization. We identified site complexity using VA’s complexity level designation criteria, with Model 1a representing the highest level of complexity, followed by Model 1b, Model 1c, Model 2 and Model 3.

#### Missing data

We encountered missing data for the following variables: sex, race-ethnicity, marital status and site complexity. For sex and site complexity, the numbers of Veterans with missing data totaled < 1% (2 Veterans were missing sex and 49 were missing site complexity data); therefore, we did not recode missing values and these observations were dropped in regression analyses. Since 9% of Veterans in our total sample were missing race-ethnicity values and 2.3% were missing marital status, we created a separate “unknown” category for each of these variables and retained these observations in our regression analyses. There were no missing values for regression outcomes (i.e., health services utilization). Regression analyses therefore included 9,000 Veterans (of 9,051) comparing CTI vs. non-CTI and 2,008 Veterans (of 2,022) comparing adequate vs. inadequate fidelity.

### Analyses

We conducted bivariate analyses of demographics, diagnoses, health services utilization and VA facility complexity among CTI vs. non-CTI Veterans; and, after restricting analyses to CTI Veterans, among Veterans at sites with adequate vs. inadequate fidelity, using t-tests for continuous variables and chi-square tests for categorical variables. In multivariate regression, we used a generalized estimating equations model with clustering by site to identify the association between CTI (vs. non-CTI) and health services utilization, as well as the association between CTI fidelity (adequate vs. inadequate) and health services utilization among CTI Veterans; we used negative binomial regression for count outcomes (number of primary care, ED, behavioral health, homeless, outpatient medical-surgical visits) and logistic regression for binary outcomes (any inpatient, inpatient mental health, or inpatient medical-surgical admission). We tested an approach using inverse probability weighting to adjust for potential confounding; however, this approach did not significantly improve the robustness of our regression models. We adjusted for Veteran predisposing characteristics (demographics), the enabling factor of PACT empanelment in the year prior to entry, evaluated need (the presence or absence of any mental health diagnosis, the presence or absence of any SUD diagnosis, multi-morbidity), and site complexity. We constructed two-tailed p-values, which were considered significant at < 0.05, and did not adjust for multiple comparisons. All analyses were conducted using Stata v19.5 [34].

## Results

### Sample characteristics

Of the 9,051 Veterans in our sample, 3,005 (33.2%) were exposed to CTI. In bivariate analyses (Table 1), CTI Veterans were more frequently Non-Hispanic White (47.2% vs. 42.0%), Hispanic (5.7% vs. 5.3%), Non-Hispanic Other Race (5.0% vs. 3.7%), and Non-Hispanic Unknown Race (10.0% vs. 8.6%) compared to non-CTI Veterans. A higher percentage of CTI Veterans lived in rural/highly rural/island areas compared to non-CTI Veterans (17.3% vs. 12.9%). CTI Veterans did not differ significantly from non-CTI Veterans with respect to sex, age, or marital status. In terms of enabling factors, CTI Veterans were less frequently PACT empaneled compared to non-CTI Veterans (81.3% vs. 86.3%), although no significant differences were observed in the proportion of Veterans empaneled in HPACT. In factors associated with evaluated need, CTI Veterans had a lower mean multi-morbidity than non-CTI Veterans, both when MWI included mental health and SUD conditions (10.7 vs. 12.2) and when these conditions were excluded (8.7 vs. 9.9). CTI Veterans had a lower prevalence of any mental health diagnosis (48.9% vs. 58.1%)—including major depressive disorder (26.2% vs. 31.9%), anxiety (20.6% vs. 23.9%), and post-traumatic stress disorder (21.8% vs. 26.7%)—and a lower prevalence of having any SUD diagnosis (27.2% vs. 35.8%)—including alcohol use disorder (18.6% vs. 26.1%), opioid use disorder (5.2% vs. 6.4%), cannabis use disorder (10.0% vs. 13.0%), sedative use disorder (0.5% vs. 1.2%), cocaine use disorder (6.7% vs. 13.0%) and other psychoactive substance use disorder (4.5% vs. 6.8%). All differences were statistically significant at the *p* < 0.05 level.

**Table 1:** Characteristics of CTI vs. Non-CTI Veterans

	CTI	Non-CTI	*p*-value	Total
**N**	3005 (32.2%)	6046 (66.8%)		9051
**Predisposing Characteristics**				
Age, M (SD)	56.0 (14.0)	56.5 (13.7)	0.095	56.4 (13.8)
Sex			0.660	
Male	2651 (88.3%)	5356 (88.6%)		8007 (88.5%)
Female	352 (11.7%)	690 (11.4%)		1042 (11.5%)
Race/Ethnicity			< 0.001	
Non-Hispanic White	1418 (47.2%)	2537 (42.0%)		3955 (43.7%)
Non-Hispanic Black	964 (32.1%)	2446 (40.5%)		3410 (37.7%)
Hispanic	172 (5.7%)	319 (5.3%)		491 (5.4%)
Non-Hispanic Other Race	150 (5.0%)	225 (3.7%)		375 (4.1%)
Non-Hispanic Unknown Race	301 (10.0%)	519 (8.6%)		820 (9.1%)
Marital Status			0.120	
Separated/Divorced	1387 (46.2%)	2822 (46.7%)		4209 (46.5%)
Married	533 (17.7%)	966 (16.0%)		1499 (16.6%)
Never Married/Single	895 (29.8%)	1896 (31.4%)		2791 (30.8%)
Widowed	112 (3.7%)	232 (3.8%)		344 (3.8%)
Unknown	78 (2.6%)	130 (2.2%)		208 (2.3%)
Residence			< 0.001	
Urban	2485 (82.7%)	5265 (87.1%)		7750 (85.6%)
Rural/Highly Rural/Islands	520 (17.3%)	781 (12.9%)		1301 (14.4%)
**Enabling Factors**				
PACT Empanelment 1 Year Pre-Entry	2444 (81.3%)	5218 (86.3%)	< 0.001	7662 (84.7%)
HPACT Empanelment 1 Year Pre-Entry	73 (2.4%)	143 (2.4%)	0.880	216 (2.4%)
**Evaluated Need**				
Multimorbidity Weighted Index (MWI)*				
MWI, M (SD)	10.7 (10.8)	12.2 (11.4)	< 0.001	11.7 (11.2)
Adapted MWI, M (SD)	8.7 (10.2)	9.9 (10.8)	< 0.001	9.5 (10.6)
Mental Health Diagnoses 1 Year Pre-Entry				
Any mental health disorder	1468 (48.9%)	3510 (58.1%)	< 0.001	4978 (55.0%)
Schizophrenia	141 (4.7%)	318 (5.3%)	0.260	459 (5.1%)
Bipolar	214 (7.1%)	486 (8.0%)	0.130	700 (7.7%)
Other psychotic disorder	94 (3.1%)	177 (2.9%)	0.600	271 (3.0%)
Major depressive disorder	786 (26.2%)	1931 (31.9%)	< 0.001	2717 (30.0%)
Anxiety	620 (20.6%)	1447 (23.9%)	< 0.001	2067 (22.8%)
Post-traumatic stress disorder (PTSD)	654 (21.8%)	1614 (26.7%)	< 0.001	2268 (25.1%)
Substance Use Disorder (SUD) Diagnoses 1 Year Pre-Entry				
Any SUD	818 (27.2%)	2166 (35.8%)	< 0.001	2984 (33.0%)
Alcohol use disorder	559 (18.6%)	1581 (26.1%)	< 0.001	2140 (23.6%)
Opioid use disorder	156 (5.2%)	389 (6.4%)	0.019	545 (6.0%)
Cannabis use disorder	301 (10.0%)	788 (13.0%)	< 0.001	1089 (12.0%)
Sedative use disorder	15 (0.5%)	70 (1.2%)	0.002	85 (0.9%)
Cocaine use disorder	202 (6.7%)	787 (13.0%)	< 0.001	989 (10.9%)
Other stimulant use disorder	193 (6.4%)	442 (7.3%)	0.130	635 (7.0%)
Inhalant use disorder	5 (0.2%)	5 (0.1%)	0.320	10 (0.1%)
Hallucinogen use disorder	11 (0.4%)	20 (0.3%)	0.850	31 (0.3%)
Other psychoactive drug use disorder	136 (4.5%)	410 (6.8%)	< 0.001	546 (6.0%)
**Health Behaviors 1 Year Post-Entry^**				
Primary care (any)	1790 (59.6%)	4236 (70.1%)	< 0.001	6026 (66.6%)
Primary care count, M (SD)	5.3 (6.2)	5.6 (6.1)	0.030	5.5 (6.1)
Emergency Department (ED, any)	775 (25.8%)	2211 (36.6%)	< 0.001	2986 (33.0%)
ED count, M (SD)	2.9 (3.6)	3.0 (3.7)	0.442	3.0 (3.7)
Outpatient mental health (any)	944 (31.4%)	2462 (40.7%)	< 0.001	3406 (37.6%)
Outpatient mental health count, M (SD)	7.3 (9.7)	7.5 (10.7)	0.612	7.4 (10.4)
Outpatient SUD (any)	192 (6.4%)	646 (10.7%)	< 0.001	838 (9.3%)
Outpatient SUD count, M (SD)	13.9 (21.8)	14.2 (21.2)	0.871	14.1 (21.3)
Outpatient behavioral health◊ (any)	999 (33.2%)	2637 (43.6%)	< 0.001	3636 (40.2%)
Outpatient behavioral health◊, M (SD)	9.5 (14.2)	10.4 (16.3)	0.162	10.2 (15.8)
Homeless services visit (any)	1059 (35.2%)	2652 (43.9%)	< 0.001	3711 (41.0%)
Homeless services count, M (SD)	5.5 (7.5)	6.6 (8.2)	<0.001	6.3 (8.1)
Outpatient medical-surgical (any)	1023 (34.0%)	2512 (41.5%)	< 0.001	3535 (39.1%)
Outpatient medical-surgical count, M (SD)	3.5 (3.8)	4.0 (4.2)	0.002	3.9 (4.1)
Inpatient hospitalization (any)	316 (10.5%)	944 (15.6%)	< 0.001	1260 (13.9%)
Inpatient count, M (SD)	2.0 (2.2)	2.1 (1.9)	0.843	2.1 (2.0)
Inpatient mental health (any)	105 (3.5%)	352 (5.8%)	< 0.001	457 (5.0%)
Inpatient mental health count, M (SD)	2.2 (2.1)	1.7 (1.2)	0.009	1.8 (1.4)
Inpatient medical-surgical (any)	202 (6.7%)	596 (9.9%)	< 0.001	798 (8.8%)
Inpatient medical-surgical count, M (SD)	1.7 (1.4)	1.9 (1.9)	0.334	1.8 (1.7)
**Facility-Level Factors**				
Site Complexity Level			< 0.001	
Model 1a	1105 (37.0%)	2720 (45.2%)		3825 (42.5%)
Model 1b	830 (27.8%)	1408 (23.4%)		2238 (24.9%)
Model 1c	206 (6.9%)	880 (14.6%)		1086 (12.1%)
Model 2	296 (9.9%)	380 (6.3%)		676 (7.5%)
Model 3	552 (18.5%)	625 (10.4%)		1177 (13.1%)

* Adapted MWI excludes mental health and substance use disorder diagnoses

^ Utilization counts were calculated among Veterans with *≥* 1 visit of the corresponding visit type in the year post-GPD entry

◊ Behavioral health includes any mental health or substance use visit

CTI Veterans also differed significantly from non-CTI Veterans in health services utilization within the year after GPD program entry. Compared to non-CTI Veterans, CTI Veterans had lower rates of health services utilization for any ambulatory health services use (i.e., any outpatient visit); considering count outcomes, CTI Veterans had fewer mean visits in primary care (5.3 vs. 5.6), homeless services (5.5 vs. 6.6), and outpatient medical-surgical visits (3.5 vs. 4.0) but higher rates of psychiatric hospitalizations (2.2 vs. 1.7). CTI Veterans more frequently received care at Model 1b (27.8% vs. 23.4%), Model 2 (9.9% vs. 6.3%) and Model 3 sites (18.5% vs. 10.4%) compared to non-CTI Veterans, who more frequently received care at sites with the highest complexity level (Model 1a).

Among CTI Veterans (*n* = 2,022), Table 2 presents characteristics of Veterans at sites with adequate (12 sites) vs. inadequate fidelity (6 sites). Nearly two-thirds of CTI Veterans (65.8%) were associated with sites that had adequate fidelity. In terms of predisposing characteristics, Veterans at sites with adequate fidelity were more frequently Non-Hispanic White (46.0% vs. 42.5%), Non-Hispanic Black (34.6% vs. 33.7%), Hispanic (6.5% vs. 3.6%) and Non-Hispanic Unknown Race (10.1% vs. 9.7%) compared to Veterans at sites with inadequate fidelity. Veterans at adequate-fidelity sites were more frequently separated/divorced (47.4% vs. 43.7%) and never married/single (30.8% vs. 27.1%) compared to Veterans at inadequate sites. Assessing enabling factors, no statistically significant differences were observed in Veterans’ PACT or HPACT empanelment in the year prior to Aftercare entry. With respect to evaluated need, we observed no significant differences between Veterans at adequate vs. inadequate sites in multi-morbidity, mental health or SUD diagnoses. Regarding health behaviors, Veterans at sites with adequate fidelity had higher rates of primary care use in the year post Aftercare-entry (61.2% vs. 56.0%) compared to Veterans at sites with inadequate fidelity. Veterans at sites with adequate fidelity also had fewer mean ED visits (2.7 vs. 3.7) but more mean homeless services visits (5.9 vs. 4.7) compared to Veterans at inadequate sites. Examining facility-level factors, Veterans at adequate sites were more frequently located at Model 1b (43.1% vs. 15.0%), Model 1c (6.1% vs. 2.9%), and Model 2 (8.0% vs. 5.4%) facilities compared to Veterans at inadequate sites.

**Table 2:** Characteristics of CTI Veterans by fidelity

	Adequate	Inadequate		*p*-value		Total
**N Sites**	12 (66.7%)	6 (33.3%)				18
**N Veterans**	1331 (65.8%)	691 (34.2%)				2022
**Predisposing Characteristics**						
Age, M (SD)	55.6 (14.0)	55.4 (14.1)		0.760		55.6 (14.0)
Sex				0.067		
Male	1192 (89.7%)	601 (87.0%)				1793 (88.8%)
Female	137 (10.3%)	90 (13.0%)				227 (11.2%)
Race/Ethnicity				< 0.001		
Non-Hispanic White	612 (46.0%)	294 (42.5%)				906 (44.8%)
Non-Hispanic Black	460 (34.6%)	233 (33.7%)				693 (34.3%)
Hispanic	87 (6.5%)	25 (3.6%)				112 (5.5%)
Non-Hispanic Other Race	37 (2.8%)	72 (10.4%)				109 (5.4%)
Non-Hispanic Unknown Race	135 (10.1%)	67 (9.7%)				202 (10.0%)
Marital Status				< 0.001		
Separated/Divorced	631 (47.4%)	302 (43.7%)				933 (46.1%)
Married	217 (16.3%)	150 (21.7%)				367 (18.2%)
Never Married/Single	410 (30.8%)	187 (27.1%)				597 (29.5%)
Widowed	52 (3.9%)	27 (3.9%)				79 (3.9%)
Unknown	21 (1.6%)	25 (3.6%)				46 (2.3%)
Residence				0.160		
Urban	1119 (84.1%)	564 (81.6%)				1683 (83.2%)
Rural/Highly Rural/Islands	212 (15.9%)	127 (18.4%)				339 (16.8%)
**Enabling Factors**						
PACT Empanelment 1 Year Pre-Entry	1071 (80.5%)	564 (81.6%)		0.550		1635 (80.9%)
HPACT Empanelment 1 Year Pre-Entry	37 (2.8%)	14 (2.0%)		0.370		51 (2.5%)
**Evaluated Need**						
Multimorbidity Weighted Index (MWI)*						
MWI, M (SD)	10.4 (10.8)	10.6 (11.1)		0.650		10.5 (10.9)
Adapted MWI, M (SD)	8.4 (10.2)	8.7 (10.5)		0.570		8.5 (10.3)
Mental Health Diagnoses 1 Year Pre-Entry						
Any mental health disorder	646 (48.5%)	315 (45.6%)		0.220		961 (47.5%)
Schizophrenia	67 (5.0%)	27 (3.9%)		0.270		94 (4.6%)
Bipolar	99 (7.4%)	39 (5.6%)		0.140		138 (6.8%)
Other psychotic disorder	47 (3.5%)	19 (2.7%)		0.430		66 (3.3%)
Major depressive disorder	355 (26.7%)	172 (24.9%)		0.390		527 (26.1%)
Anxiety	278 (20.9%)	129 (18.7%)		0.240		407 (20.1%)
Post-traumatic stress disorder (PTSD)	269 (20.2%)	150 (21.7%)		0.450		419 (20.7%)
Substance Use Disorder (SUD) Diagnoses 1 Year Pre-Entry						
Any SUD	352 (26.4%)	166 (24.0%)		0.260		518 (25.6%)
Alcohol use disorder	236 (17.7%)	105 (15.2%)		0.150		341 (16.9%)
Opioid use disorder	61 (4.6%)	35 (5.1%)		0.660		96 (4.7%)
Cannabis use disorder	121 (9.1%)	59 (8.5%)		0.740		180 (8.9%)
Sedative use disorder	7 (0.5%)	4 (0.6%)		1.000		11 (0.5%)
Cocaine use disorder	75 (5.6%)	47 (6.8%)		0.320		122 (6.0%)
Other stimulant use disorder	79 (5.9%)	46 (6.7%)		0.560		125 (6.2%)
Inhalant use disorder	2 (0.2%)	0 (0.0%)		0.550		2 (0.1%)
Hallucinogen use disorder	8 (0.6%)	0 (0.0%)		0.057		8 (0.4%)
Other psychoactive drug use disorder	65 (4.9%)	32 (4.6%)		0.830		97 (4.8%)
**Health Behaviors 1 Year Post-Entry^**						
Primary care (any)	815 (61.2%)	387 (56.0%)		0.025		1202 (59.4%)
Primary care count, M (SD)	5.2 (6.0)	5.0 (5.0)		0.622		5.1 (5.7)
Emergency Department (ED, any)	378 (28.4%)	180 (26.0%)		0.270		558 (27.6%)
ED count, M (SD)	2.7 (2.6)	3.7 (5.7)		0.003		3.0 (3.9)
Outpatient mental health (any)	421 (31.6%)	207 (30.0%)		0.450		628 (31.1%)
Outpatient mental health count, M (SD)	7.3 (10.0)	7.5 (9.4)		0.806		7.4 (9.8)
Outpatient SUD (any)	73 (5.5%)	49 (7.1%)		0.170		122 (6.0%)
Outpatient SUD count, M (SD)	15.0 (26.9)	11.6 (15.7)		0.472		13.7 (23.2)
Outpatient behavioral health◊ (any)	442 (33.2%)	222 (32.1%)		0.650		664 (32.8%)
Outpatient behavioral health◊, M (SD)	9.4 (15.2)	9.5 (12.9)		0.903		9.4 (14.5)
Homeless services visit (any)	483 (36.3%)	224 (32.4%)		0.085		707 (35.0%)
Homeless services count, M (SD)	5.9 (7.6)	4.7 (5.2)		0.031		5.5 (7.0)
Outpatient medical-surgical (any)	493 (37.0%)	227 (32.9%)		0.063		720 (35.6%)
Outpatient medical-surgical count, M (SD)	3.4 (3.6)	3.6 (4.2)		0.509		3.5 (3.8)
Inpatient hospitalization (any)	151 (11.3%)	72 (10.4%)		0.550		223 (11.0%)
Inpatient count, M (SD)	1.9 (1.8)	2.5 (2.9)		0.088		2.1 (2.2)
Inpatient mental health (any)	40 (3.0%)	29 (4.2%)		0.200		69 (3.4%)
Inpatient mental health count, M (SD)	1.8 (1.3)	2.6 (2.1)		0.083		2.1 (1.7)
Inpatient medical-surgical (any)	110 (8.3%)	44 (6.4%)		0.130		154 (7.6%)
Inpatient medical-surgical count, M (SD)	1.8 (1.4)	1.9 (1.7)		0.739		1.8 (1.5)
**Facility-Level Factors**						
Site Complexity Level				< 0.001		
Model 1a	472 (35.6%)	266 (38.8%)				738 (36.7%)
Model 1b	571 (43.1%)	103 (15.0%)				674 (33.5%)
Model 1c	81 (6.1%)	20 (2.9%)				101 (5.0%)
Model 2	106 (8.0%)	37 (5.4%)				143 (7.1%)
Model 3	94 (7.1%)	260 (37.9%)				354 (17.6%)

* Adapted MWI excludes mental health and substance use disorder diagnoses

^ Utilization counts were calculated among Veterans with *≥* 1 visit of the corresponding visit type in the year post-GPD entry

◊ Behavioral health includes any mental health or substance use visit

### Regression analyses

In multivariate regression analyses, CTI Veterans had lower utilization rates for all health service types compared to their non-CTI counterparts (Table 3), including primary care visits (Incidence Rate Ratio [IRR] = 0.98, 95% CI: 0.97–0.98), ED visits (IRR = 0.93, 95% CI: 0.90–0.95), outpatient behavioral health visits (IRR = 0.97, 95% CI: 0.97–0.98), homeless services visits (IRR = 0.91, 95% CI: 0.89–0.93), and outpatient medical-surgical visits (IRR = 0.94, 95% CI: 0.92–0.96). In addition, CTI Veterans had lower likelihood of having any inpatient hospitalization (Adjusted Odds Ratio [AOR] = 0.79, 95% CI: 0.68–0.91), any inpatient mental health hospitalization (AOR = 0.75, 95% CI: 0.60–0.95) and any inpatient medical-surgical hospitalization (AOR = 0.82, 95% CI: 0.68–0.98), compared to non-CTI Veterans.

**Table 3:** Incidence rate ratios and adjusted odds ratios for CTI veterans (*n* = 3005) compared to non-CTI veterans (*n* = 6046) for counts of outpatient utilization visits and likelihood of any inpatient stay estimated using adjusted generalized estimating equations with negative binomial distribution*

Utilization Type	IRR	*p*-value	95% CI
Primary care	0.98	< 0.001	0.97–0.98
Emergency Department	0.93	< 0.001	0.90–0.95
Outpatient behavioral health	0.97	< 0.001	0.97–0.98
Outpatient homeless services	0.91	< 0.001	0.89–0.93
Outpatient specialty medical-surgical	0.94	< 0.001	0.92–0.96
	**AOR**	***p-value***	**95% CI**
Inpatient hospitalizations (all)	0.79	0.002	0.68–0.91
Inpatient mental health	0.75	0.018	0.60–0.95
Inpatient medical-surgical	0.82	0.028	0.68–0.98

IRR = Incident Rate Ratio, AOR = Adjusted Odds Ratio, CI = Confidence Interval

* Adjusting for age, sex, race/ethnicity, marital status, urban-rural residence, PACT empanelment, mental health diagnoses, SUD diagnoses, multi-morbidity, and site complexity

Among CTI Veterans, we compared utilization rates by fidelity status (Table 4). CTI Veterans at sites with adequate fidelity had higher rates of primary care use (IRR = 1.04, 95% CI: 1.02–1.07), lower rates of ED visits (IRR = 0.84, 95% CI: 0.80–0.90) and higher rates of homeless services use (IRR = 1.10, 95% CI: 1.05–1.15) compared to CTI Veterans at sites with inadequate fidelity. No statistically significant differences were observed in outpatient behavioral health visits, outpatient medical-surgical visits, and inpatient hospitalizations of any type.

**Table 4:** Incidence rate ratios and adjusted odds ratios for veterans at sites with adequate fidelity (*n* = 1331) compared to veterans at sites with inadequate fidelity (*n* = 691) for counts of outpatient utilization visits and likelihood of any inpatient stay estimated using adjusted generalized estimating equations with negative binomial distribution*

Utilization Type	IRR	*p*-value	95% CI
Primary care	1.04	< 0.001	1.02–1.07
Emergency Department	0.84	< 0.001	0.80–0.90
Outpatient behavioral health	1.01	0.186	0.99–1.03
Outpatient homeless services	1.10	< 0.001	1.05–1.15
Outpatient specialty medical-surgical	1.03	0.320	0.97–1.08
	**AOR**	***p-value***	**95% CI**
Inpatient hospitalizations (all)	1.14	0.454	0.80–1.63
Inpatient mental health	0.62	0.081	0.36–1.06
Inpatient medical-surgical	1.49	0.068	0.97–2.29

IRR = Incident Rate Ratio, AOR = Adjusted Odds Ratio, CI = Confidence Interval

* Adjusting for age, sex, race/ethnicity, marital status, urban-rural residence, PACT empanelment, mental health diagnoses, SUD diagnoses, multi-morbidity, and site complexity

## Discussion

We conducted secondary analyses of administrative data from a CTI implementation initiative in community-based organizations that deliver case management to Veterans. Specifically, we studied the impacts of CTI on VA health services utilization in a population of homeless-experienced VA users, and associations between health services utilization and adequate versus inadequate fidelity. Though we hypothesized that CTI Veterans would have increased ambulatory care use and decreased acute care use versus their non-CTI counterparts, we found that CTI and non-CTI Veterans had similar service use patterns, with slightly lower utilization rates among CTI Veterans across all service types. With regards to implementation fidelity, these data support our hypothesis that Veterans at sites with adequate fidelity had higher rates of using primary care and homeless services; however, contrary to our hypothesis, we found no significant differences in other types of ambulatory care use (e.g., outpatient behavioral health or medical-surgical visits).

The inconclusive impacts of CTI on health services utilization may point to challenges coordinating VA care in a non-VA context (i.e., Aftercare agencies that partner with VA but are community-based). In other studies, these homeless service providers have reported limited knowledge about the VA healthcare system and inadequate communication as barriers to coordinating VA care for Veterans [35]. Similarly, homeless-experienced Veterans who use both VA and community-based services are more likely to report unfavorable care coordination experiences (e.g., perceived delays) compared to those who exclusively use VA [36]. Strengthening linkages between community partners and VA healthcare could improve our observed outcomes as well as care experiences for homeless-experienced Veterans more generally. Moreover, although some studies have identified a reduction in total nights hospitalized among CTI recipients compared to those who did not receive CTI [16, 37], our findings are consistent with more recent literature in which differences between CTI and non-CTI recipients in health services utilization outcomes—including acute and outpatient care—were non-significant [38].

Nevertheless, these analyses highlight the importance of assessing practice fidelity when studying effectiveness in implementation trials. Without examining CTI fidelity, one could assume that the minimal differences in health service utilization between CTI and non-CTI Veterans reflect the limited impacts of CTI on this outcome. However, the significant differences in health services utilization among CTI Veterans at sites with adequate versus inadequate fidelity speak to the value of examining implementation fidelity when studying the effectiveness of evidence-based practices in implementation trials.

Our finding that Veterans at sites with adequate implementation fidelity had decreased use of ED services (versus Veterans at sites with inadequate CTI fidelity) was expected, particularly alongside our findings of increased primary care use among Veterans at sites with adequate fidelity. There is significant literature to suggest that primary care engagement reduces acute care utilization [39, 40]. At the VA, primary care is often considered an entry point to the healthcare system, facilitating connections to other types of services [41, 42]; thus, we were surprised that the increased use of primary care and homeless services observed among Veterans at sites with adequate fidelity did not translate into increased utilization of behavioral health services. However, we note that behavioral health services may have unique access barriers, including patients’ fear of stigmatization or negative perceptions of care [43–45]. Additionally, the support provided by CTI may have addressed Veterans’ needs for which behavioral health services are typically sought.

We also note that our findings with regards to fidelity-focused analyses may be explained by site-specific factors, as opposed to fidelity to CTI alone. Our CTI implementation sites included a mix of high-performing and low-performing sites; it is unclear whether the high-performing sites were those with adequate fidelity to CTI or whether other contextual characteristics (e.g., interdisciplinary care coordination) led to improved care utilization at these sites (and contributed to fidelity ratings). Evaluating the impacts of fidelity may require a more nuanced understanding of site-level contextual characteristics and their interplay with implementation fidelity [46, 47].

Our work points to the need for further investigation into the relationship between CTI fidelity and health services use outcomes. Qualitative research with Veterans who were exposed to CTI may provide additional insight into their relationships with CTI case managers and how CTI may have impacted their perceptions of VA services as well as their VA service use patterns. Additionally, an alternative study design, incorporating pre-intervention data, could produce more robust comparisons of CTI vs. non-CTI, and adequate vs. inadequate fidelity, groups.

### Limitations

This study has several limitations. Our findings are specific to homeless-experienced Veterans enrolled in VA healthcare and limited to VA service use collected from VA administrative data; they may not be generalizable to non-Veteran populations or to homeless-experienced Veterans who do not use VA healthcare. We were also limited by the use of site complexity as our only site-level variable; our analyses may have been enhanced with additional contextual data on participating sites, including site-level factors that may have contributed to differences in health services utilization outside the context of CTI implementation. In addition, our data on need were collected from VA administrative data encounter diagnosis coding and not from chart review; however, we applied best practices to identify records, including counting at least two diagnosis records from outpatient visits or one record from inpatient admission. We collapsed racial and ethnic information to improve regression results; as a result, we were not able to assess the effects of race and ethnicity more granularly.

Moreover, our sites were not randomized to CTI versus no CTI conditions; rather, sites were selected to implement CTI because of perceived readiness or need for improved services. As a result of our non-random study design, causal inference was limited, making it difficult to assess whether the observed effects of CTI (or fidelity), on health services use are attributable to CTI (or fidelity) or to other pre-existing differences between Veterans. Our analyses are therefore applicable only to the period after CTI delivery and should not be interpreted to predict health service use over time.

As a sensitivity analysis, we estimated the same outcome models using inverse probability of treatment weights. Results were substantively similar to the covariate-adjusted estimates, suggesting that our observed covariates were sufficient to address confounding; however, estimators remained susceptible to bias from unmeasured site-level confounders, as mentioned above. Alternatively, incorporating pre-intervention data may have addressed concerns about pre-existing differences in healthcare utilization behavior between Veterans at CTI vs. non-CTI sites (or sites with adequate vs. inadequate fidelity). Our study was limited by the lack of access to pre-implementation data at the site level. Future evaluations would benefit from a difference-in-difference analysis across the cohorts to identify changes in healthcare utilization over time.

## Conclusions

Identifying the impacts of CTI on Veterans’ health services utilization requires attention to additional mediating factors, such as the adequacy of implementation fidelity. When implemented with adequate fidelity, CTI may reduce Veterans’ utilization of costly ED services. Ensuring efforts to improve fidelity to CTI implementation may hold value in promoting optimal care linkages for homeless-experienced Veterans and other clients with high health service needs.

## Data Availability

The raw datasets underlying this publication are not available due to data privacy laws. Participant data cannot be shared outside of the VA due to VA policies designed to protect participants’ privacy. For more information, please contact the lead author, Kimberly Clair: [kimberly.clair@va.gov].

## Abbreviations

AOR: Adjusted Odds Ratio
CAN: Care Assessment Need
CDW: VA Corporate Data Warehouse
CTI: Critical Time Intervention
ED: Emergency Department
EHR: Electronic Health Record
GPD: Grant and Per Diem
HPACT: Homeless patient–aligned care team
ICD: International classification of diseases
IRB: Institutional Review Board
IRR: Incidence Rate Ratio
MDD: Major Depressive Disorder
MWI: Multimorbidity–Weighted Index
PACT: Patient–aligned care team
PTSD: Post–traumatic stress disorder
QUERI: Quality Enhancement and Research Initiative
SSN: Social Security Number
SUD: Substance Use Disorder
VA: United States Department of Veterans Affairs
